# Role of regulation of PD-1 and PD-L1 expression in sepsis

**DOI:** 10.3389/fimmu.2023.1029438

**Published:** 2023-03-09

**Authors:** Teng Zhang, Li Yu-Jing, Tao Ma

**Affiliations:** ^1^ Department of General Surgery, Tianjin Medical University General Hospital, Tianjin, China; ^2^ Department of Neurology, Tianjin Medical University General Hospital, Tianjin, China

**Keywords:** PD-1, PD-L1, sepsis, immune cells, immunology

## Abstract

Long term immunosuppression is problematic during sepsis. The PD-1 and PD-L1 immune checkpoint proteins have potent immunosuppressive functions. Recent studies have revealed several features of PD-1 and PD-L1 and their roles in sepsis. Here, we summarize the overall findings of PD-1 and PD-L1 by first reviewing the biological features of PD-1 and PD-L1 and then discussing the mechanisms that control the expression of PD-1 and PD-L1. We then review the functions of PD-1 and PD-L1 in physiological settings and further discuss PD-1 and PD-L1 in sepsis, including their involvement in several sepsis-related processes and their potential therapeutic relevance in sepsis. In general, PD-1 and PD-L1 have critical roles in sepsis, indicating that their regulation may be a potential therapeutic target for sepsis.

## 1 Introduction

Sepsis is a severe illness caused by an aberrant host response to infections, and it is associated with acute organ failure and a high mortality risk (1). Although there has been a global improvement in clinical outcomes as a result of improved treatment practices resulting from the dissemination and implementation of the Surviving Sepsis Campaign guidelines (2) over the preceding decades (3), mortality rates remain unacceptably high, ranging from 25 to 30 percent for sepsis and 40 to 50 percent in cases of septic shock, with country-specific variations (4–6). Moreover, many sepsis survivors have long-term physical and cognitive impairments as well as higher death rates than the general population (7–11).

Years ago, it was believed that sepsis mortality and morbidity resulted from an excessive systemic inflammatory response, but medications designed to reduce this response did not enhance survival (12, 13). Several investigations have shown that sepsis is not only characterized by early acute inflammation but is also a concomitant immunosuppressed condition that may last for months after the original episode of sepsis (14, 15). Immune suppression during sepsis makes it harder to eliminate the underlying infection and increases the chance of subsequent infections (16, 17). Importantly, the chronic immunosuppressed states generated by defective innate and adaptive immune responses are responsible for reduced immunity, multi-organ damage, protracted hospital stays, and mortality (17–21). To properly treat this condition, it is essential to understand how sepsis produces immunosuppression.

Immune checkpoint pathways are endogenous immune system components that govern the immune response under normal physiological conditions (22). The programmed cell death protein 1 (PD-1) and programmed death ligand 1 (PD-L1) immune checkpoint is an important regulator that inhibits T cell receptor-induced activation signals (23). In addition, interaction between PD-L1 and PD-1 suppresses the immune system systemically in many cells (24, 25). Multiple clinical studies have established a correlation between PD-1 or PD-L1 expression and sepsis mortality (26–28). Immunotherapy for sepsis using anti-PD-1 and anti-PD-L1 antibodies has shown benefit in animal studies (29–32) while clinical trials in humans have not given the direct benefit evidence of PD-1 or PD-L1 blockade (33, 34). The purpose of this review is to describe the biological properties of PD-1 and PD-L1 and their functions in physiological conditions, focusing on the mechanisms that regulate PD-1 and PD-L1 expression and the roles of the PD-1/PD-L1 axis in sepsis. In general, PD-1 and PD-L1 have critical roles in sepsis, indicating that regulation of their expression may be a potential therapeutic target for sepsis.

## 2 PD-1 and PD-L expression and structure

PD-1, also known as CD279, is one of the co-inhibitory receptors initially found on the surface of antigen-activated T lymphocytes (35). A small percentage of lymph node, spleen, and bone marrow cells, as well as immature CD4+CD8+ thymocytes has been reported to express the PD-1 protein (36). The presence of PD-1 (mRNA or protein) is seldom detected and appears only after a period of stimulation (37). Activation of lymphocyte B cell receptors or T cell receptors is often associated with an increase in PD-1 expression (38, 39).

PD-L1 (CD274) and PD-L2 (CD273) are the two ligands for PD-1 (CD279). PD-L1 expression can be found on hematopoietic cells, such as T lymphocytes, B lymphocytes, macrophages and dendritic cells (DCs), as well as non-hematopoietic healthy tissue cells, such as vascular endothelial cells, keratinocytes, pancreatic islet cells, astrocytes, corneal epithelial cells, and endothelial cells (40). It has been reported that macrophages, DCs, and mast cells express PD-L2 (41). Binding to PD-L1 is the major mechanism of PD-1 function in sepsis (42). Moreover, PD-L1 gene deficiency improves sepsis survival, while PD-L2 gene deficiency does not show a survival benefit for sepsis (43).

**Figure 1:**
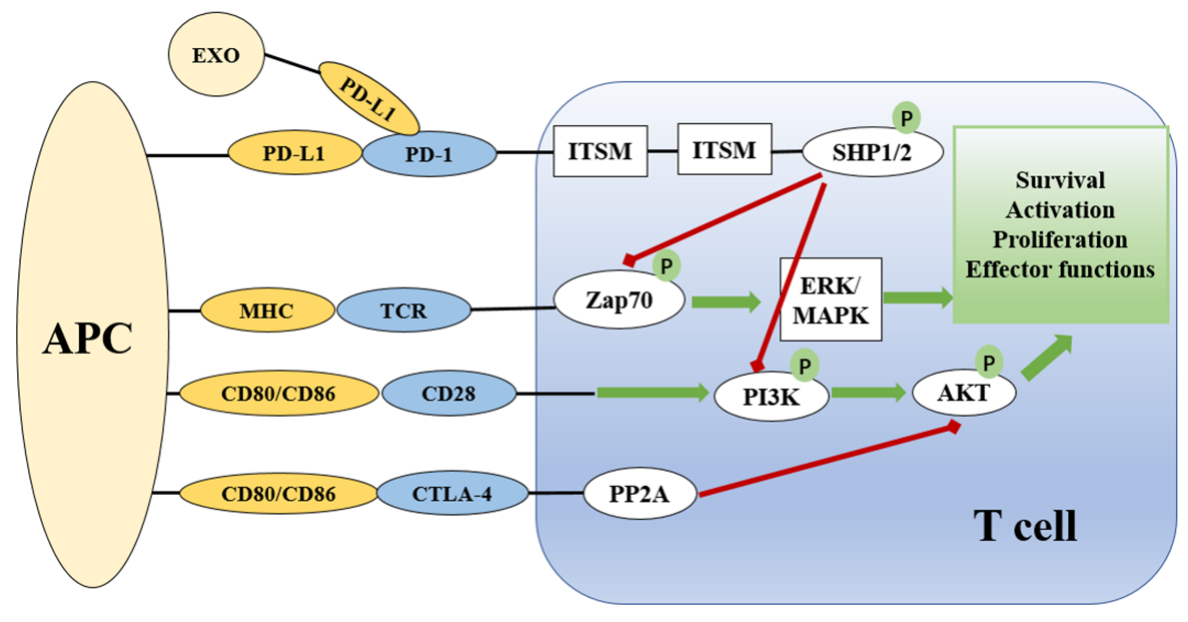
Overview of the PD-1 and PD-L1 checkpoints and signaling pathways associated with them. The presentation of antigen by MHC on APCs to the TCR complex on T cells activates T cells *via* the Zap70 and ERK/MAPK signaling pathways. CD28 on T cells binds to CD80/86 on APCs to provide co-stimulatory signals. PD-1/PD-L and CTLA-4 signaling suppress the AKT signaling pathway to limit T cell activation. CTLA-4 suppresses the AKT pathway directly by recruiting PP2A, while PD-1 signaling includes SHP-mediated regulation of Zap20 and the PI3K/AKT signaling pathway. Green lines indicate stimulatory messages, while red lines represent inhibitory ones. ITSM and ITIM are intracellular domains of immunological checkpoints that are responsible for intracellular signaling. APC, antigen presenting cell; TCR, T cell receptor; MHC, major histocompatibility complex; PD-1, programmed death-1; CTLA4, cytotoxic T lymphocyte antigen-4; ZAP70, zeta chain of T cell receptor associated protein kinase 70; PI3K, phosphoinositide 3 kinase; PP2A, protein phosphatase 2A; ERK, extracellular signal-regulated kinase; MAPK, mitogen activated protein kinase; AKT, protein kinase B; ITIM, immunoreceptor tyrosine-based inhibition motif; ITSM, immunoreceptor tyrosine-based motif; SHP, Src homology region 2 domain-containing phosphatase.

Both PD-1 and PD-L1 are type I transmembrane immunoglobulin (Ig) superfamily members (41). PD-1 contains a cytoplasmic domain that comprises two tyrosine-based signaling motifs and an extracellular domain that mimics Ig-V as well as a transmembrane domain (40). PD-L1 has an Ig-V extracellular domain, an Ig-C-like extracellular domain, a transmembrane domain, and a short cytoplasmic tail devoid of conventional signaling patterns (44). It is possible for the extracellular domains of PD-L1 and PD-1 to interact, causing PD-1 to alter its shape, which allows Src family kinases to phosphorylate the immuno-receptor tyrosine-based inhibitory motif (ITIM) and immuno-receptor tyrosine-based switch motif (ITSM) (45) (**Figure 1**). These phosphorylated tyrosine patterns attract the SHP-2 and SHP-1 protein tyrosine phosphatases, which suppress the activation of T lymphocytes (46). When the PD-1 receptor is ligated, SHP-2 inhibits the Akt and ERK/MAPK signaling pathways by dephosphorylating PI3K (47). In the absence of SHP-2-induced T cell exhaustion, SHP-1 plays a compensatory role (48). In addition, the SHP2 phosphatase is capable of dephosphorylating the CD28 costimulatory receptor (44).

## 3 Regulation of PD-1 and PD-L1 expression

### 3.1 Regulation of PD-1 expression

The mechanisms that regulate PD-1 expression in T cells are well known. PD-1 is barely detectable on naive T cells, but PD-1 surface expression rapidly increases on all T cells upon first antigen-mediated activation through the T cell receptor (TCR) (49). When the activating antigen is rapidly eliminated, PD-1 expression levels on responding T cells decrease (50, 51). If the antigen is not eliminated, such as during persistent infections and malignancies, PD-1 expression persists at a high level (50–52). PD-1 expression on antigen-activated T cells is controlled by many transcription factors, including nuclear factor of activated T cells (NFAT), cytoplasmic 1, fork head box protein O1 (FOXO1), T-bet, and B lymphocyte-induced maturation protein 1 (Blimp-1) (40, 53), as well as the serine–threonine kinase glycogen synthase kinase 3 (GSK3) (54). Although TCR activation is the most essential factor in controlling T cell PD-1 expression, other factors independent of TCR activation also play a role. For instance, in chronic infection, PD-1 expression may be sustained even after antigen clearance (55–57). The following re-expansion of exhausted CD8+ T cell populations under infection also persistently express PD-1 (56). There are dynamic patterns of DNA methylation at the Pdcd1 gene that correspond with PD-1 expression during T cell development (58). Using assay for transposase-accessible chromatin with sequencing (ATAC-seq), researchers identified a distinct pattern of accessibility of the Pdcd1 gene in fatigued T cells (57, 59), and ablation of a regulatory region 23 kb upstream of the transcriptional start site decreases PD-1 expression (59). This 23 kb upstream region in mouse T cells is essential for regulating PD-1 expression (59).

### 3.2 Regulation of PD-L1 expression

**Figure 2:**
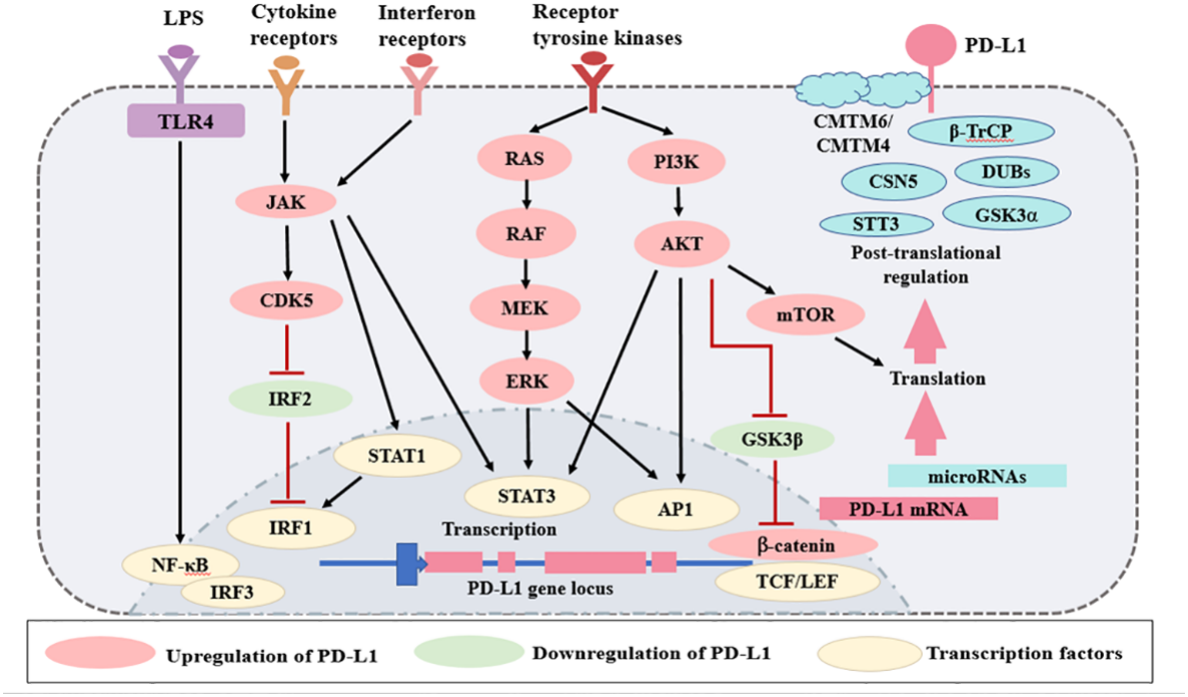
Regulation of PD-L1 expression. The expression of PD-L1 is regulated by several mechanisms. Various signaling pathways, such as TLRs, interferon receptors, cytokine receptors, and receptor tyrosine kinases, increase PD-L1 expression. There are several microRNAs that regulate PD-L1 mRNA transcription. Finally, PD-L1 is regulated at the protein level by protein circulation, ubiquitination, and glycosylation. Red lines represent inhibitory signals, and black lines represent stimulatory signals. AP1, activator protein 1; LPS, lipopolysaccharides; LEF, lymphoid enhancer- binding factor; TLRs, Toll-like receptors; NF- κB, nuclear factor κB; IRF, interferon regulatory factor; STAT, signal transducer and activator of transcription; TCF, T cell- specific transcription factor; GSK3, glycogen synthase kinase 3; JAK, Janus kinase; CDK5, cyclin dependent kinase 5; CMTM, CKLF-like MARVEL transmembrane domain containing; DUBs, deubiquitinating enzymes; STT3, subunit of the oligosaccharyltransferase complex; GSK3α, glycogen synthase kinase 3α; β-TrCP, β-tranducin repeat-containing protein.

In contrast to PD-1, PD-L1 is ubiquitously expressed by several kinds of cells and regulated by more factors in an inflammation environment. There are three major regulatory mechanisms of PD-L1 expression (**Figure 2**) as follows: 1) proinflammatory signals promote the expression of PD-L1; 2) microRNAs control the post-transcriptional regulation of PD-L1 gene expression; 3) protein circulation, ubiquitination, and glycosylation all influence PD-L1 levels. The regulators of PD-L1 expression have been listed in **Table 1**.

**Table 1:** Regulators of PD-L1 in normal cells.

Type	Regulators	Tissue type
**Inflammatory** signaling	IFN-γ	↑endothelial cells (60), monocytes (61), dendritic cells, macrophages (61, 62), renal tubular epithelial cells (63), and neutrophils (64)
	IFN-α and IFN-β	↑endothelial cells (65), monocytes and dendritic cells (66)
	TLR4	↑macrophages (67), monocytes (68), and dendritic cells (69)
	TLR3	↑dendritic cells (70) and endothelial cells (71)
	TNF-α	↑endothelial cells (60), dendritic cells (72), and monocytes (73, 74)
	TGF-β	↑dendritic cells (75, 76) and T cells (77) ↓renal tubular epithelial cells (78) and monocytes (74)
	IL-6	↑dendritic cells (72)
	IL-10	↑dendritic cells (79) and monocytes (73)
	IL-1β	↑dendritic cells (72)
	IL-17	↑monocytes (73)
	IL-12	↑endothelial cells (65) and monocyte-derived macrophages (80)
	IL-27	↑dendritic cells (81, 82)
	HIF1α	↑myeloid cells (83)
**microRNA**	miR-513	↓cholangiocytes (84, 85)
	miR-155	↓dermal lymphatic endothelial cells (86)
	miR-200	↓cancer cells (87)
	miR-34	↓cancer cells (88)
Protein level **regulation**	CMTM6	↑dendritic cells (89, 90)
	β-catenin	↑dendritic cells (91)
	GSK3β	↓cancer cells (92, 93)
	E3 ligases	↓cancer cells (94)
	CSN5	↑cancer cells (95)

“↑” means up-regulate PD-L1 expression, and “↓” means down-regulate PD-L1 expression.

#### 3.2.1 Inflammatory signaling mediates PD-L1 regulation

PD-L1 gene expression is linked to inflammation, which is consistent with its role in preventing T cell activation (96). Numerous soluble chemicals generated by immune cells have recently been discovered as PD-L1-inducing agents. IFN-γ is traditionally considered to be the most potent soluble inducer of PD-L1, indicating that PD-L1 expression may be a crude indicator of IFN-γ signaling and T cell activation in the majority of instances (97). In sepsis, activated T cells produce significant levels of the IFN-γ proinflammatory cytokine (98). Upon binding to its receptor, IFN-γ activates the JAK-STAT pathway, which in turn activates the STAT1 protein (99), resulting in an increase in a group of transcription factors known as interferon-responsive factors (100), which increase the induction of PD-L1 (101). In addition to IFN-γ, type I interferons (IFN-α and IFN-β) may stimulate PD-L1 expression in endothelial cells, monocytes, and DCs *in vitro* (65, 66). Type I and type II interferons may activate the AKT-mTOR cascade, which regulates interferon-dependent mRNA translation (102), indicating that the interferon receptor signaling pathway and the AKT-mTOR signaling pathway interact (103). The phosphatidylinositol 3-kinase (PI3K) signaling pathway influences cell growth and survival (104), and pharmacological inhibition of PI3K-AKT signaling inhibits IFN-induced PD-L1 expression (100). Additionally, the PI3K-AKT pathway may regulate PD-L1 expression in an IFN-independent manner, and it has been proposed that at least a part of this regulation occurs *via* altering PD-L1 mRNA levels by mTOR (105).

Lipopolysaccharide (LPS) is the principal component of the outer membrane of Gram-negative bacteria, contributing significantly to the structural integrity of the bacterium and protecting it against certain forms of chemical attack (106). LPS treatment of macrophages (67), monocytes (68), and primary bone marrow-derived DCs (69) results in enhanced PD-L1 expression. LPS signals *via* Toll-like receptor 4 (TLR4), and activation of nuclear factor kappa-light-chain-enhancer of activated B cells (NF-kB) leads to the generation of type I interferons (107, 108). In addition, RELA, an NF-kB subunit, assembles into a complex with the PD-L1 promoter called RELA-MUC1-C, which in turn increases transcription of PD-L1 (109). Polyinosinic: polycytidylic acid [poly(I:C)] is an immunostimulant that is used to simulate viral infections (110). Poly(I:C) induces TLR3 activation on DCs and endothelial cells, thereby increasing PD-L1 expression (111), and this process requires PI3K signaling for the increase of PD-L1 expression (112).

Hypoxia is a critical feature of sepsis, as impaired lung function and drastic inflammation often outgrows the oxygen supply. Immune cells respond to this oxygen deficiency by activating a series of hypoxia-inducible factors (HIFs) (113). Both HIF-1a and HIF-2a have been shown to physically interact with the hypoxia response element (HRE) in the promoter region of PD-L1 (83, 114).

Furthermore, it has been shown that the expression of PD-L1 may be regulated by other stimulators. *In vitro* cultivated monocytes and tubular epithelial cells express less PD-L1 when treated with transforming growth factor (TGF)-β (115). PD-L1 expression in endothelial cells may be stimulated by IL-12 (116) and tumor necrosis factor (TNF) (117). When stimulated with IL-2, IL-17, IL-15 (118), IL-12 (116), IL-4, and granulocyte-macrophage colony-stimulating factor (GM-CSF) (119), monocytes and macrophages display higher amounts of surface PD-L1expression. DCs treated with IL-1, IL-6, IL-10, IL-27 (120), and TNF (117) exhibit elevated levels of PD-L1. Despite the fact that the aforementioned findings reveal that a large range of inflammatory mediators may regulate PD-L1 expression, it remains unclear in many situations whether this control occurs indirectly, such as through influencing IFN production.

#### 3.2.2 MicroRNA-mediated PD-L1 regulation

MicroRNAs play an important role in normal physiology as posttranscriptional gene expression regulators by controlling the degradation of target mRNA and/or inhibiting translation. A recent study has demonstrated the role of microRNAs in the control of PD-L1 expression (121), which may take place either directly by binding to PD-L1 mRNA or indirectly by regulating the expression of other PD-L1 regulators. Traditionally, miR-513 and miR-155 are mechanisms for fine-tuning PD-L1 expression in response to IFN-signaling. Both miR-513 and miR-155 suppress PD-L1 at the translational level by direct binding to the 3’ UTR of PD-L1 mRNA (84, 122). IFN-γ suppresses miR-513 expression while reinforcing PD-L1 expression, whereas IFN-γ induces miR-155 while suppressing PD-L1 expression (86). In addition to these direct effects, miRNAs may also indirectly affect PD-L1 expression by influencing the expression of other PD-L1 regulators, such as by repressing PTEN (a tumor-suppressor gene that negatively regulates PI3K-AKT signaling) to increase PD-L1 expression, or by inhibiting PD-L1 expression *via* its direct action on the STAT3 transcription factor (123, 124).

#### 3.2.3 PD-L1 regulation at the protein level

Expression of PD-L1 is ultimately controlled by posttranslational regulation. Autophagy and endocytosis require lysosomal breakdown to recycle cytoplasmic proteins, organelles, extracellular proteins, and cell surface receptors. CKLF-like MARVEL transmembrane domain-containing 6 (CMTM6) is a transmembrane protein that interacts with the PD-L1 protein on the cell surface (89). CMTM6 binds to PD-L1 and extends its half-life by blocking ubiquitination and lysosomal degradation during protein recycling (89). These effects increase and sustain elevated levels of PD-L1 on the cell surface (90). Inhibition of CMTM6 expression reduces PD-L1 protein synthesis, but it has little effect on PD-L1 mRNA levels (69).

PD-L1 has four residues, namely, N35, N192, N200, and N219, which are attached by an oligosaccharide (92). This N-linked glycosylation is essential for PD-L1 stability and PD-L1 binding ability (92, 125). β-catenin enhances PD-L1 glycosylation and stabilization by increasing transcription of the STT3 subunit (N-glycosyltransferase component) of the oligosaccharyltransferase complex (126, 127). Unglycosylated PD-L1 is a fragile protein (92). GSK3β phosphorylates residues T180 and S184 of PD-L1, which are subsequently bound by the β-tranducin repeats containing protein (β-TrCP) E3 ubiquitin ligase and then targeted for ubiquitin-dependent degradation by the 26S proteasome (92, 93). Glycosylation at N192, N200, and N219 impairs the interaction with GSK3β and stabilizes PD-L1 as a result (92). The phosphorylation and degradation of PD-L1 by GSK3β is a crucial mechanism for decreasing PD-L1 levels.

E3 ligases perform a critical function by binding ubiquitin chains to their targets, thereby designating them for degradation (128). There are several different E3 ligases that can degrade PD-L1 in both normal and diseased states (94). Deubiquitinating enzymes (DUBs) prevent substrate protein ubiquitination by removing ubiquitin chains, therefore stabilizing the protein (95). Deubiquitination mediated by COP9 signalosome 5 (CSN5) leads to TNF-induced activation of PD-L1 (95). Further, there are many other protein regulatory mechanisms of PD-L1 that have been reviewed in other articles (96).

## 4 Physiological function of the PD-1/PD-L1 pathway

PD-1 and PD-L1 are important to maintain a healthy body (129). In the absence of PD-1, excessive immune-mediated tissue damage may have catastrophic effects on the host. Different genetic backgrounds of PD-1-deficient animals are susceptible to developing lupus-like autoimmune disease (130, 131) or catastrophic autoimmune cardiomyopathy (132). PD-1 inhibition, whether genetic or antibody-based, has also been shown to accelerate the onset of diabetes in individuals who are neither obese nor diabetic (133). Other findings include the defect of T-cell training in the thymus in PD-1 deficient mice (131) and the impairment of maternal tolerance in fetuses and their mothers as a result of PD-L1 inhibition (134).

The PD-1 pathway plays an important role in limiting immunopathological responses in host tissues by promoting inflammatory response downregulation and return to immune system balance (135). If CD8+ T cell responses are not well regulated, significant immunopathology may occur from the production of proinflammatory cytokines, such as IFN-γ and TNF. Lethal immunopathology occurs in PD-1deficient or PD-L1deficient animals after infection with strains of lymphocytic choriomeningitis virus (LCMV) that produce chronic infection, illustrating the critical function of the PD-1 pathway in regulating immune-mediated tissue damage (50, 136, 137). This deadly immunopathology is based on CD8+ T cells and may involve the perforin-dependent destruction of vascular endothelial cells (136). The PD-1 pathway also regulates proatherogenic inflammatory responses because animals defective in the low-density lipoprotein receptor develop more atherosclerotic lesions if they lack PD-L1 (138). The reduced vascular integrity that occurs in the absence of PD-1 signaling provides a significant hurdle for PD-1 immunotherapy because inhibiting PD-1 may increase the risk of heart attacks, strokes, and edema by altering the permeability of the vascular barrier (138).

The PD-1 pathway also affects the development and responses of memory T cells. Compared to wild-type T cells, PD-1-deficient T cells isolated from vaccinia virus-infected mixed bone marrow chimera mice display greater amounts of CC-chemokine receptor 7 (CCR7) and CD62L, and they are skewed toward a central memory T cell phenotype (139). Experimentally induced deletion of PD-1 results in a higher proliferation of memory T cells when they are transplanted into wild-type recipients and then challenged with another strain of vaccine virus (139). Experiments using vaccinia virus infection have indicated that PD-1 inhibition during secondary challenge may repair deficiencies in CD8+ T cell responses in the absence of CD4+ T cell assistance (140). Secondary PD-1 blockage after viral lung infection significantly improved CD8+ T cell activities (141). The amount and quality of memory T cell responses may be affected by PD-1 inhibition during primary versus secondary challenges, and this may be dependent on the illness environment.

## 5 PD-1/PD-L1 axis in sepsis

Sepsis is a lethal uncontrolled host reaction to infection. Clinically, sepsis is currently defined as having an infection and a sudden change in how an organ works, as measured by the Sequential Organ Failure Assessment score (142). We still don’t know all the details of how cell damage and organ malfunction result from sepsis. Reduced T lymphocyte function, impaired myeloid cell activity, and non-immune cell death have been described as the pathophysiological features of sepsis (143).

**Figure 3:**
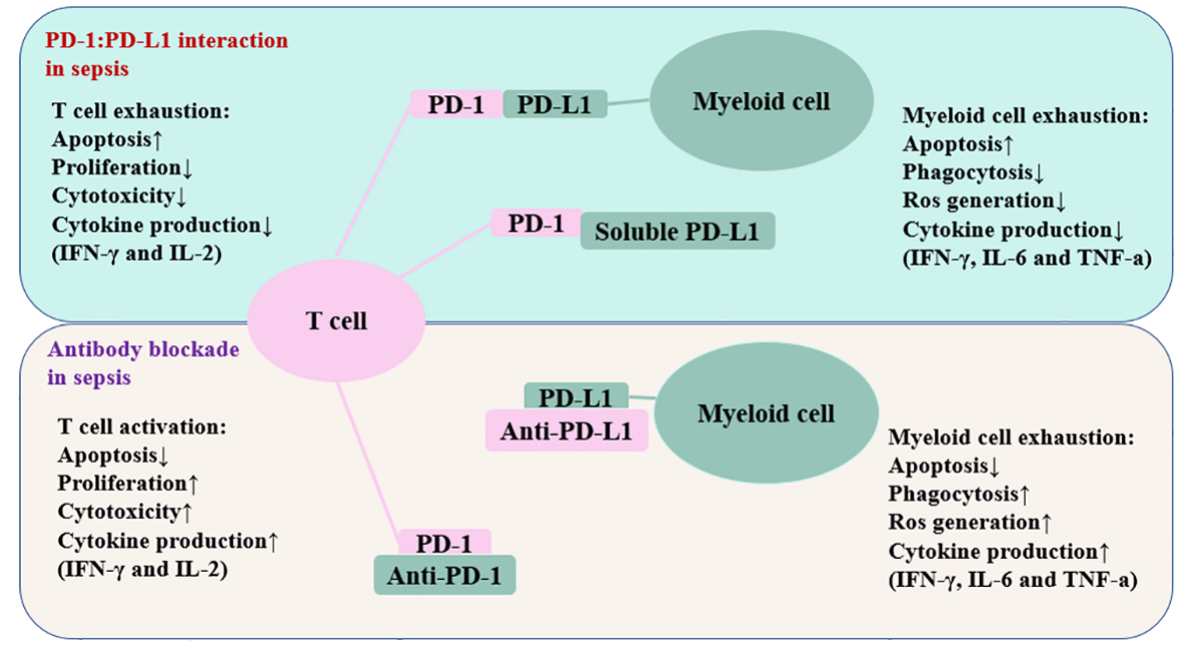
Schematic depiction of the PD-1/PD-L1 related immune cell dysfunction. The interaction between PD-1 and PD-L1 impairs T cell function and myeloid cell function. Antibodies against these inhibitory molecules restore the immune system’s function and boost resistance to infection in patients suffering from sepsis. The up arrow represents an increase, whereas the down arrow denotes a decrease. PD-1, programmed cell death-1; PD-L1, programmed cell death ligand-1; IFN-γ, interferon-gamma; IL-2, interleukin-2; IL-6, inerleukin-6.

The number of cells that express the PD-1 and PD-L1 genes is increased during sepsis (144). In the CLP model, CD4+ T cells increase PD-1 expression within 24 hours, CD8+ T cells increase PD-1 expression at a time of 3 days to 7 days, and myeloid cells increase PD-L1 expression within 24 hours (144). A previous study has reported that endothelial cells in splenic capillaries of individuals who died of sepsis had a higher level of PD-L1 than endothelial cells in the spleens of individuals with brain death or injury necessitating immediate splenectomy (27). Individuals with sepsis have increased surface PD-1 expression on T lymphocytes and increased surface PD-L1 expression on myeloid cells (28). Loss of PD-1 signaling often enhances immunological control of numerous forms of infection, such as viral, fungal, and bacterial infections (136, 141, 145–147). Numerous studies have connected PD-1/PD-L1 axis to altered immune cell activity in sepsis (**Figure 3**).

### 5.1 The PD-1/PD-L1 axis inhibits T lymphocyte function

High levels of PD-1 on T cells have been detected in patients with sepsis (148). Higher PD-1 expression on T cells has been associated with lymphopenia, T cell death, increased mortality (27, 28, 149, 150), and subsequent nosocomial infections (151). By suppressing T cell proliferation, survival, cytokine generation, and other effector activities, engagement of PD-1 by PD-L1 changes the activity of T cells in a number of ways (79, 152, 153). Binding of PD-L1 to effector T cells that express PD-1 reduces costimulatory signaling, resulting in depletion of the T cell response capacity, characterized by diminished co-stimulatory receptor expression (such as CD28), activation of inhibitory immunological checkpoints, and metabolic derangements, leading to impaired synthesis of effector cytokines, poor proliferation, and increased susceptibility to apoptotic cell death (154). In sepsis, blocking the binding of PD-1 on T cells with PD-L1 prevents T cell depletion and is associated with increased microbial clearance and a decreased mortality rate (27, 155–159).

### 5.2 The PD-1/PD-L1 axis impairs myeloid cell function

Sepsis inhibits the functionality of myeloid cells *via* the PD-1/PD-L1 axis (144). In sepsis patients, an increase in PD-L1 expression on monocytes and neutrophils is accompanied with a decrease in their phagocytic capabilities (158). It has been demonstrated that cecal ligation and puncture (CLP)-induced murine sepsis increases PD-1 expression on liver Kupffer cells and that deleting PD-1 enhances their phagocytic activity (160). In a sepsis CLP model, the expression of PD-L1 on circulating neutrophils is correlated with both pro- and anti-inflammatory cytokine levels and mortality (161). Patients with septic shock who have elevated PD-L1 levels on their circulating monocytes are more likely to die during the first 28 days of their illness (162). Recent research has shown that natural killer (NK) cell PD-L1 expression within 24 h of ICU admission is related to increased sepsis severity (163). Patients with sepsis have elevated PD-L1 levels in their peripheral blood monocytes, and binding with PD-1 impairs cell survival and function. In addition, anti-PD-1 antibody therapy restores monocyte production of critical cytokines, including IFN-γ and IL-2 (158).

### 5.3 PD-1/PD-L1 signaling causes non-immune cell death

PD-1/PD-L1 signaling has been associated with organ damage induced by sepsis (158). In addition to leukocytes, non-immune cells, such as the lung, liver, kidney, colon, small intestine, and tissue endothelial cells express PD-L1 (40, 43, 164). CLP-induced PD-L1 defective sepsis mice have lower levels of serum bilirubin, alanine aminotransferase (ALT), and aspartate aminotransferase (AST), and their endothelial permeability barrier is unaltered, with greater systemic bacteria clearance and better survival (43). Deletion of PD-L2 enhances systemic bacterial clearance but does not protect against the increase in hepatocellular damage markers, such as serum bilirubin and AST, and it does not influence the survival of CLP model (43). Both PD-1 and PD-L1 are increased on liver sinusoidal endothelial cells in a CLP model of sepsis, and deleting PD-L1 protects against sepsis-induced increases in hepatic vascular leakage, edema, and endothelial cell mortality (165). Patients with severe sepsis have PD-L1 expression in their postmortem lung tissue (27). Pulmonary endothelial cell permeability and lung damage are reduced when PD-1 or PD-1 is deleted from lung endothelial or parenchymal cells during sepsis (166, 167). Neutrophil infiltration and sepsis-induced lung damage may both be reduced by intravenous delivery of siRNAs that specifically target PD-L1 expression, indicating that this protein may be a therapeutic target for the prevention of sepsis-induced lung injury (166). PD-L1 also controls the intestinal damage caused by sepsis. Deletion or treatment with an antibody of PD-L1 lowers the degree of intestinal damage resulting from sepsis (168). In the early phase of systemic LCMV infection, PD-L1 deficiency on endothelial cell leads to increased vascular permeability and ultimately to circulatory collapse (136). It is important to note that shock or systemic inflammatory syndromes related to different pathogens might respond differently to checkpoint blockade or inhibition.

### 5.4 Therapeutic targeting of the PD-1/PD-L1 pathway

PD-1/PD-L1 signaling is a potential therapeutic target of sepsis. Patients with sepsis have been integrated using the Persistent Inflammation, Immunosuppression, and Catabolism Syndrome (PICS) (142). The inflammatory and immune-suppressing phases were considered to happen at the same time in sepsis (143). At initially, several anti-inflammatory strategies were tested since sepsis was thought to be fundamentally hyperinflammation. Yet, none of these sepsis therapy methods have shown any evidence of success (142). Therefore, immunological stimulation is a novel approach to combating sepsis, particularly its Immunoparalysis component. In CLP-induced sepsis, PD-1 knockout animals have a greater likelihood of survival than wild-type mice (169). Anti-PD-L1 antibody treatment in mouse models of CLP-induced sepsis reduces T cell apoptosis, increases bacterial clearance, and minimizes organ damage (31). Anti-PD-L1 antibody treatment 24h after fungal sepsis caused by Candida albicans protects T cell function and enhances survival (30). The two-hit model is another type of sepsis model. It has a higher death rate than the CLP model and a more severe immune suppression than the CLP model. Using a two-pronged attack, first CLP-induced sepsis and then fungal sepsis using C. albicans, researchers created a model of sepsis and found that the novel PD-L1 blocking peptide, compound 8, decreases mortality by half (170). Similarly, a recent study has reported that treating CLP-induced sepsis with the novel LD01 peptide, which suppresses PD-1 signaling, enhances macrophage phagocytosis, T cell activity, and survival (171). No single study showed benefit of PD-1/PD-L1 blockage in sepsis caused by pulmonary infection, and the majority of pre-clinical models examined did not include antibiotic treatment, which is a critical part of therapy for sepsis (172). Anti-PD-L1 therapy did not alter the survival of sepsis model caused by *Staphylococcus aureus* pneumonia (173). It should be noted that different organisms, different sites of infection, and timing of therapy all may have an effect on the outcomes of anti-PD-1 or anti-PD-L1 antibody treatment in sepsis.

For humans, most studies compared PD-1 or PD-L1 expression between sepsis patients and healthy volunteers but not critically ill patients. The effectiveness of PD-1 or PD-L1 blockade on human cells has just been tested *in vitro* blockade assays (174). Ex vivo administration with an anti-PD-1 antibody decreases apoptosis and increases IFN-γ production in CD8 T cells collected from septic patients (150) as well as reverses sepsis-induced T cell dysfunction and enhances neutrophil and monocyte phagocytic activity in circulating blood cells collected from septic patients (158). The anti-PD-1 antibody, nivolumab, has been licensed for use in cancer patients (175, 176). Clinical trials for nivolumab in sepsis have also made progress. A 2019 Phase 1b clinical trial investigating the safety and tolerability of nivolumab in septic patients reported no side effects, such as a cytokine storm (33). Similarly, another 2019 Phase 1b clinical trial investigating the safety of the anti-PD-L1 antibody, BMS-936559, in sepsis patients validated the antibody’s safety with no instances of hypercytokinemia (34). All studies gave evidence that there were no safety concerns with PD-1/PD-L1 blockade in ICU-bound sepsis patients at high risk for mortality and no indication of a “cytokine storm”. No evidence has shown the benefit of immunotherapy (PD-1 and PD-L1 blockade) in clinical trials in humans for sepsis (33, 34). Interesting, recent studies have indicated that the anti-PD-1/PD-L1 axis may be safe for use in sepsis (33, 34). However, cancer research studies have revealed a wide variety of major side effects related to immune checkpoint inhibition, including liver damage, thrombocytopenia, pneumonitis, colitis, thyroiditis, and vasculitis (177). Additional clinical trials will provide better knowledge of the blockade of the PD-1/PD-L1 axis in sepsis.

## 6 Discussion and conclusion

PD-1 and PD-L1 are potent immune checkpoint proteins in several cells, and they are upregulated by several inflammation signals. In sepsis, PD-1 and PD-L1 expression is corelated with the mortality rate, and blockade of PD-1 or PD-L1 protects against sepsis. In current state, the blockade of PD-1/PD-L1 would be able to correct the immunosuppression in sepsis, as in oncology treatment. However, many unknowns remain. First, it is unknown how PD-1 and PD-L1 differ from other inhibitory receptors in terms of their specific and shared functions. Inhibitory receptors are not known to act in a hierarchical manner; if one receptor is destroyed, other receptors may compensate. These signaling pathways are still undefined. For example, it remains unknown how blocking one receptor affects the ability of another to communicate. Second, methods are needed to inhibit PD-1 or PD-L1 expression increases in sepsis. The blockade of PD-1/PD-L1 can cause the immune system to attack healthy cells, leading to a range of immune-related adverse events such as rash, colitis, pneumonitis, and hepatitis (178). In sepsis, PD-L1 expression is increased as a result of immune dysregulation. Restoring PD-L1 expression levels to normal by inhibiting PD-L1 expression upregulation is a potential therapeutic modality capable of avoiding autoimmune damage due to PD-L1 blockade. In summary, we believe that regulating the PD-1/PD-L1 pathway will be a potent weapon to protect against sepsis in the future.

## Author contributions

TZ contributed to collection of references and manuscript preparation. TM and LY-J contributed to manuscript modifications. All authors contributed to the article and approved the submitted version.
